# Side Effects from Cancer Therapies and Perspective of 1044 Long-Term Ovarian Cancer Survivors—Results of Expression VI–Carolin Meets HANNA–Holistic Analysis of Long-Term Survival with Ovarian Cancer: The International NOGGO, ENGOT, and GCIG Survey

**DOI:** 10.3390/cancers15225428

**Published:** 2023-11-15

**Authors:** Hannah Woopen, Maren Keller, Dario Zocholl, Suzana Mittelstadt, Maria-Pilar Barretina-Ginesta, Viola Heinzelmann-Schwarz, Judith Lafleur, Roman Kocián, Joanna Baum, Petra Krabisch, Patriciu Achimas-Cadariu, Mehmet Ali Vardar, Ignace Vergote, Sara Nasser, Theresa Link, Marta Gil-Martin, Tibor A. Zwimpfer, Katharina Leitner, Marcin Jedryka, Tamara Boxler, Elena Ioana Braicu, Jalid Sehouli

**Affiliations:** 1 Department of Gynecology with Center for Oncological Surgery, Corporate Member of Freie Universität Berlin and Humboldt-Universität zu Berlin, Charité—Universitätsmedizin Berlin, 13353 Berlin, Germany; 2 North-Eastern German Society for Gynecological Oncology (NOGGO), 13359 Berlin, Germany; 3 Institute of Biometry and Clinical Epidemiology, Corporate Member of Freie Universität Berlin and Humboldt-Universität zu Berlin, Charité—Universitätsmedizin Berlin, 10117 Berlin, Germany; 4 Department of Women’s Health, University Hospital Tübingen, 72076 Tübingen, Germany; 5 Institut Català d’Oncologia, Medical Oncology Department, 17007 Girona, Spain; 6 Precision Oncology Group, Institut d’Investigació Biomèdica de Girona, 17007 Girona, Spain; 7 Medical Sciences Department, Universitat de Girona, 17003 Girona, Spain; 8 Grupo Español de Investigación en Cáncer de Ovario (GEICO), 28003 Madrid, Spain; 9 Department of Gynecology and Gynecologic Oncology, University Hospital of Basel, 4056 Basel, Switzerland; 10 Swiss GO Trial Group (Swiss-GO), 4031 Basel, Switzerland; 11 Department of Gynecology and Obstetrics, Ordensklinikum Barmherzige Schwestern Linz, 4020 Linz, Austria; 12 Arbeitsgemeinschaft Gynaekologische Onkologie Austria (AGO Austria), 6020 Innsbruck, Austria; 13 Department of Gynaecology, Obstetrics and Neonatology First Faculty of Medicine, Charles University and General University Hospital in Prague, 12 108 Prague, Czech Republic; 14 General University Hospital in Prague, 12 808 Prague, Czech Republic; 15 Central and Eastern European Gynecologic Oncology Group (CEEGOG), 128 51 Prague, Czech Republic; 16 Department of Gynaecology and Obstetrics, Klinikum Chemnitz, 09116 Chemnitz, Germany; 17 Institute of Oncology Prof. Dr. I. Chiricuta Cluj-Napoca, University of Medicine and Pharmacy Iuliu Hatieganu, 400015 Cluj-Napoca, Romania; 18 Department of Gynecologic Oncology, Cukurova University, 01250 Adana, Turkey; 19 Turkish Society of Gynecologic Oncology (TRSGO), Ovecler, 06450 Ankara, Turkey; 20 Division of Gynaecological Oncology, Leuven Cancer Institute, University Hospitals Leuven, 3000 Leuven, Belgium; 21 Belgium and Luxembourg Gynaecological Oncology Group (BGOG), 3000 Leuven, Belgium; 22 Pan-Arabian Research Society of Gynecological Oncology (PARSGO), 13353 Berlin, Germany; 23 Department of Gynecology and Obstetrics, Technische Universität Dresden, 01307 Dresden, Germany; 24 Catalan Institute of Oncology—IDIBELL, L’Hospitalet-Barcelona, 08908 Barcelona, Spain; 25 Department of Obstetrics and Gynecology, Innsbruck Medical University, 6020 Innsbruck, Austria; 26 Gynecological Oncology Department, Wroclaw Medical University, 50-367 Wroclaw, Poland; 27 Oncological Gynecology Department, Lower Silesian Oncology, Pulmonology and Hematology Center, 53-413 Wroclaw, Poland; 28 Frauenklinik Fürth, 90766 Fürth, Germany

**Keywords:** survivorship: long-term survivors, ovarian cancer, follow-up care, long-term side effects

## Abstract

**Simple Summary:**

The aim of this survey of long-term survivors (LTS) with ovarian cancer was to enhance the knowledge on the patient characteristics and health concerns of this growing patient cohort in order to tailor follow-up care. Follow-up care is usually carried out within the first five years after initial diagnosis, before they are transferred to general physicians and gynecologists. Interestingly, in our cohort of 1044 LTS from 14 countries, most patients with a median survival time of eleven years after initial diagnosis still received regular follow-up care (94.2%). Forty-six percent still reported cancer- and therapy-related symptoms (i.e., gastrointestinal problems, fatigue, lymphedema, and neurological problems). The present analysis confirms the symptom checklist and clinical relevance of the GCIG consensus guideline on long-term survival with gynecological cancer. Follow-up care should be offered beyond the typical five years as specialized survivorship care with a focus on long-term side effects, lifestyle, and prevention.

**Abstract:**

The aim of this survey was to increase the knowledge on the characteristics and health concerns of long-term survivors (LTS; survival > 5 years) after ovarian cancer in order to tailor follow-up care. This international survey was initiated by the NOGGO and was made available to members of ENGOT and GCIG. The survey is anonymous and consists of 68 questions regarding sociodemographic, medical (cancer) history, health concerns including distress, long-term side effects, and lifestyle. For this analysis, 1044 LTS from 14 countries were recruited. In total, 58% were diagnosed with FIGO stage III/IV ovarian cancer and 43.4% developed recurrent disease, while 26.0% were receiving cancer treatment at the time of filling in the survey. LTS who survived 5–10 years self-estimated their health status as being significantly worse than LTS who survived more than 10 years (*p* = 0.034), whereas distress also remained high 10 years after cancer diagnosis. Almost half of the cohort (46.1%) reported still having symptoms, which were mainly lymphedema (37.7%), fatigue (23.9%), pain (21.6%), polyneuropathy (16.9%), gastrointestinal problems (16.6%), and memory problems (15.5%). Almost all patients (94.2%) regularly received follow-up care. Specialized survivorship care with a focus on long-term side effects, lifestyle, and prevention should be offered beyond the typical five years of follow-up care.

## 1. Introduction

Among all malignant gynecological tumors, ovarian cancer has the highest mortality rates with a 5-year survival rate of 30–40%. Late first diagnosis in the majority of cases at clinical tumor stage III or IV account for these mortality rates. Nevertheless, advanced oncological surgery with radical cytoreductive operation methods, the centralization of patient treatment, and progress in adjuvant platinum-based chemotherapy and maintenance therapy have reduced these mortality rates over recent decades. Since the introduction of targeted therapies about 10 years ago, an increasing number of patients survive more than 5 years and now up to 31% are long-term survivors (LTS). As a consequence, medical caretakers now face a rising number of ovarian cancer LTS who survive disease-free or with active or recurrent disease [1]. A growing number of survivors receive long-term treatment including targeted therapies [2,3,4]. This is reflected by the recent definition of long-term survival with ovarian cancer with a survival of at least five years after initial diagnosis, independently from the current status of the disease and history of recurrences [5]. To date, prospective data to evaluate prognostic factors for long-term survival are scarce. However, from large retrospective population-based studies, some factors have been identified as being associated with long-term survival, such as younger age at diagnosis, earlier clinicopathological stage, lower grade, non-serous histology, BRCA status, immune profile, mutational signatures, absence of ascites, optimal cytoreduction at primary surgery, duration of survival after a recurrence, and response to subsequent chemotherapy or surgery [6,7,8,9,10,11,12]. Follow-up after cancer is usually concentrated on the first five years after the initial diagnosis, with a focus on recognizing the recurrence of cancer. After these five years, patients are generally no longer seen by (gynecologic) oncologists, but are often referred to general physicians and gynecologists instead. Long-term concerns are not evaluated systematically, and symptoms may not be recognized as being tumor- or cancer-treatment-related anymore. However, long-term survivors (LTS) with ovarian cancer still experience a wide range of symptoms, like fatigue or neurological toxicity, that influence their quality of life [13,14,15,16,17]. The side effects occurring during or shortly after chemotherapy regimens are well known. However, there are only limited data available regarding the long-term side effects or late-onset side effects. To the best of our knowledge, there is no large multicenter cohort available involving long-term survivors with ovarian cancer. The aim of this prospective study was, therefore, (1) to characterize long-term survivors with ovarian cancer, (2) to analyze the main medical concerns within and after the first five years after initial diagnosis, and (3) to improve follow-up care.

## 2. Materials and Methods

For the international survey ‘Expression VI—Long-term survival with ovarian cancer’, long-term survivors (LTS) with ovarian cancer were recruited within the study ‘Carolin meets HANNA—Holistic Analysis of loNg-term survivors with ovariaN cAncer’ since November 2016. At the beginning of the study, long-term survival was defined as survival of at least eight years after initial diagnosis and was adapted in July 2020 to the proposed definition of long-term survival provided by the Gynecologic Cancer InterGroup (GCIG) as survival of at least five years after initial diagnosis. The aim of the survey was to identify the needs and expectations of long-term survivors and enhance the clinical knowledge on this patient cohort.

The study was initiated by the North-Eastern German Society of Gynecological Oncology (NOGGO) and the Department of Gynecology in the Center for Oncological Surgery of the Charité—Universitätsmedizin Berlin. Many of the questions derive from prior large ‘Expression surveys’ initiated by the NOGGO. The questionnaire was tested using ten long-term survivors for readability, comprehension, and feasibility. The survey ‘Expression VI’ was then reviewed and made available to members of ENGOT (European Network of Gynaecological Oncological Trial Groups) and GCIG (Gynecologic Cancer InterGroup). The survey was translated by certified translators in respective national languages and validated by bilingual physicians. Ethical approval was obtained from local ethics committees, with the leading ethics committee being the ‘Charité—Universitätsmedizin Berlin’ in Germany (AVD-No: EA2/139/16).

The survey was available both online (available in German and English on the website www.carolinmeetshanna.com) and as a paper-based questionnaire (available in Arabic, Czech, Dutch, English, French, German, Hungarian, Italian, Polish, Romanian, Slovakian, Slovenian, Spanish, Turkish, and Ukrainian). The survey was available in the following 14 countries: Austria (AGO Austria—Arbeitsgemeinschaft Gynaekologische Onkologie Austria), Belgium (BGOG—Belgium and Luxembourg Gynaecological Oncology Group), Czech Republic (CEEGOG—Central and Eastern European Gynecologic Oncology Group), Germany (NOGGO—North-Eastern German Society of Gynecological Oncology), Hungary (CEEGOG), Poland (CEEGOG), Romania, Saudi Arabia (PARSGO—Pan-Arabian Research Society of Gynecological Oncology), Slovakia (CEEGOG), Slovenia (CEEGOG), Spain (GEICO—Grupo Español de Investigación en Cáncer de Ovario), Switzerland (Swiss GO Trial Group), Turkey (TRSGO—Turkish Society of Gynecologic Oncology), and Ukraine (CEEGOG).

The inclusion criteria were as follows:

- Women ≥ 18 years with a history of ovarian, tubal, or peritoneal cancer with an initial diagnosis of at least five years.
- Willingness to take part in the survey.

There were no exclusion criteria. Recurrences and active cancer treatment did not exclude participants. The survey was distributed to potential participants by the study centers and study groups. Our cohort was recruited from both hospitals and local gynecological practices. Patients were asked to participate during regular follow-up visits, which are offered every six to twelve months on a regular basis in order to detect potential recurrent disease as opposed to presentation due to a specific symptom. The definition of long-term survival also applies to patients undergoing current treatment so that the survey was not restricted to routine follow-up patients, but also included patients undergoing current cancer treatment. Information about the Expression VI survey was also promoted in patient conferences, self-help-groups, and patient journals.

The survey was anonymous. A patient consent form was not needed as it was assumed that only patients who would like to participate would take part. The questionnaire consisted of 68 questions. The questions were mainly closed-ended with dichotomous, multiple choice, rank order, or rating questions, but there were also a few open-ended questions. Apart from information about patient characteristics (i.e., age, stage, cancer treatment, recurrences, medical and social history), the patients were asked about their current symptoms, side effects during treatment, long-term toxicities, and follow-up care. Health competence was also evaluated in regard to nutrition, physical activity, faith, and mental health (distress thermometer). The whole questionnaire can be viewed on the survey’s website (www.carolinmeetshanna.com).

All statistical analyses were performed with the program R 4.2.1 (R = Foundation for Statistical Computing, Vienna, Austria). The analysis was mainly descriptive. Continuous data were described using mean, median, range, and standard deviation. Categorical data were described using absolute frequencies and percentages. For statistical comparisons between groups, the Mann–Whitney U test was used for all continuous variables, and the Chi-squared test or Fisher’s exact test for categorical variables. All hypothesis tests were purely explorative, and *p*-values below 0.05 were considered statistically significant.

## 3. Results

Between November 2016 and February 2021, a total of 1044 long-term survivors (LTS) with ovarian cancer were recruited from 14 countries. The median age was 68 years (patients’ characteristics are shown in Table 1).

The median survival time at recruitment was 11 years (range: 5–46 years). At the time of inclusion, 308 women (29.5%) had survived 5–10 years and 736 women (70.5%) had already survived more than 10 years. More than half of all participants with known FIGO stage were diagnosed with ovarian cancer at an advanced stage (24.8% FIGO I, 16.6% FIGO II, 58.6% FIGO III/IV). At initial diagnosis, 98.6% received surgery followed by chemotherapy in 92.0%. Gastrointestinal resection was performed in 11.2% (*n* = 123), and partial liver resection in 1.7% (*n* = 18). Half of the patients (49.1%, *n* = 513) received lymphonodectomy, which did not differ between patients with early- and advanced-stage ovarian cancer (FIGO I/II: 59.4% vs. FIGO III/IV: 64.8%; *p* = 0.22). Almost half of all patients developed recurrent disease (43.4%). Out of 930 patients who provided information on whether they were undergoing treatment at the time of recruitment, 12.7% stated that they were under current cancer treatment.

### 3.1. Health Status and Long-Term Side Effects

**Figure 1:**
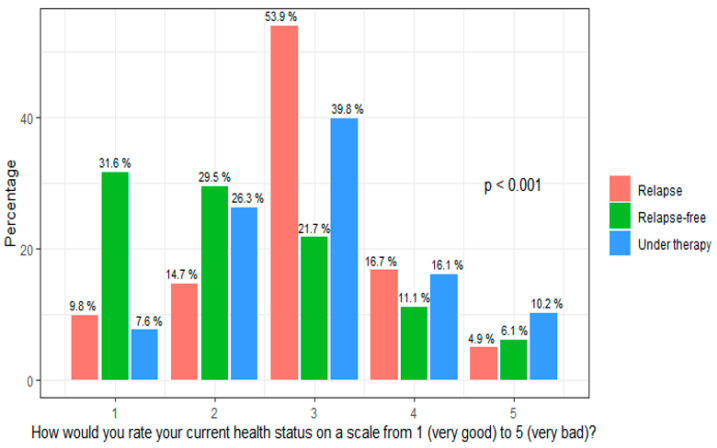
Health status in association with current cancer treatment and relapse: long-term survivors who received cancer treatment at recruitment and who developed recurrent disease had a worse health status.

Health status was rated very good (=1) or good (=2) by 52.0% of the participants, while 20.3% reported a bad (=4) or very bad (=5) health status. A moderate health status (=3) was reported by 27.7%. Health status was rated significantly worse by patients with recurrent disease, with a mean value of 2.74 (*p* < 0.001), and by patients under current cancer treatment, with a mean value of 2.95 vs. 2.34 in patients without recurrent disease (Figure 1).

**Figure 2:**
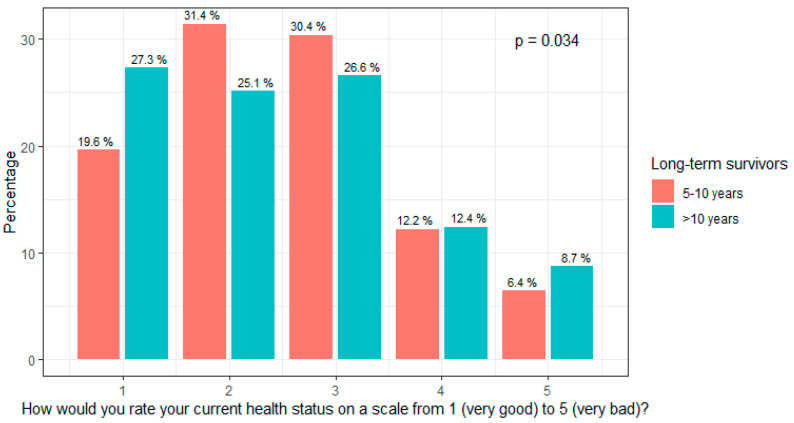
Health status in association with survival years. Survival years have an impact on health status. Patients who survived more than ten years reported a better health status (*p* = 0.034).

There was no difference in the health status between patients with early-stage and advanced-stage ovarian cancer (*p* = 0.39). The median health status was better in patients after lymphadenectomy (*p* = 0.002). The health status was self-estimated as being significantly worse in LTS 5–10 years after diagnosis than in LTS > 10 years after diagnosis (*p* = 0.034, Figure 2), whereas there was no difference in the perception of distress.

**Figure 3:**
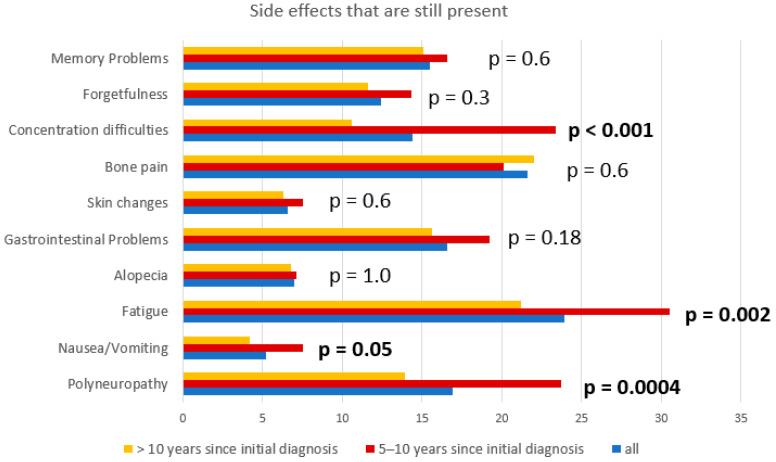
Side effects and symptoms in association with survival years. A total of 55.2% of LTS who survived 5–10 years and 42.1% of LTS who survived >10 years reported still having symptoms. Fatigue, polyneuropathy, nausea, and concentration difficulties improved with the time of survival.

In terms of symptoms, 55.2% of LTS who survived 5–10 years and 42.1% of those who survived >10 years reported still having symptoms. Symptoms were more frequently seen in patients with a history of recurrent disease compared to patients without recurrence at recruitment (58.4% vs. 36.5%, *p* < 0.001). Younger patients (≤60 years) reported more frequent medical symptoms compared to LTS > 60 years (51.1% vs. 42.8%, *p* = 0.01). The most frequently reported symptoms were lymphedema (37.7%), fatigue (23.9%), pain (21.6%), polyneuropathy (16.9%), gastrointestinal problems (16.6%), and memory problems (15.5%). Fatigue, polyneuropathy, nausea, and concentration difficulties improved with the time of survival. However, 21.2% still suffered from fatigue, 13.9% from polyneuropathy, and 10.6% from concentration difficulties. Figure 3 depicts the side effects that were still seen in LTS according to survival years. Interestingly, long-term survivors with fatigue described more frequent long-term side effects like polyneuropathy, cognitive disorders, depression, and gastrointestinal disorders compared to those without fatigue (*p* < 0.001). Fatigue, memory problems, concentration difficulties, and distress were more frequently seen in younger patients (*p* = 0.06, *p* < 0.001, *p* = 0.001, and *p* = 0.002). Elderly patients tended to report more gastrointestinal problems (*p* = 0.06). Patients with early-stage ovarian cancer (FIGO I/II) at initial diagnosis reported fatigue, bone pain, and gastrointestinal problems less frequently than patients with advanced-stage ovarian cancer (18.4% vs. 31.4%; *p* = 0.0011; 15.0% vs. 24.6%; *p* = 0.009, and 12.1% vs. 21.5%; *p* = 0.0064). Lymphadenectomy was associated with lymphedema, fatigue, concentration difficulties, and polyneuropathy (*p* < 0.001, *p* = 0.03, *p* = 0.03, and *p* < 0.001, respectively).

**Figure 4:**
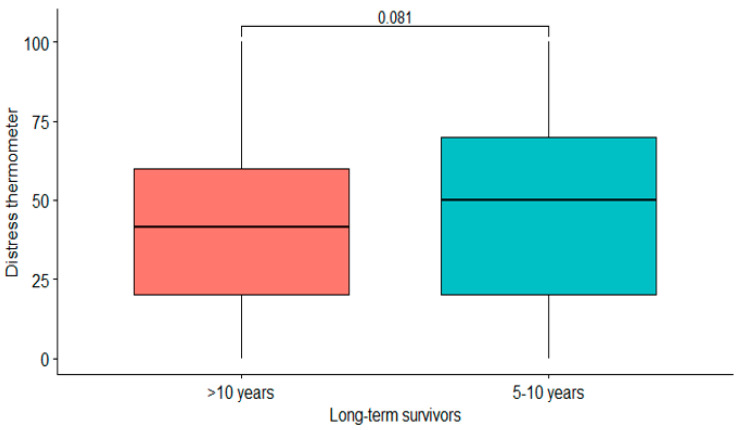
Distress according to survival years. Survival years did not influence distress: *p* = 0.081.

The median distress level assessed using the distress thermometer was 50 (range: 0–100). Relapse and current cancer treatment at recruitment were associated with higher distress levels (*p* < 0.001 and *p* = 0.002). There was no significant difference regarding the amount of distress in LTSs with a survival time of 5–10 years compared to those with a survival time > 10 years (*p* = 0.081) (Figure 4). Early-stage and advanced-stage ovarian cancer patients showed similar distress levels (*p* = 0.26). Patients after lymphonodectomy did not show higher distress levels (*p* = 0.31). In this cohort, 42.8% still regarded themselves as cancer patients. This was more often the case among LTS with recurrent disease compared to those without recurrent disease (67.8% vs. 22.4%, *p* < 0.001). A total of 48.7% of LTS who survived their cancer diagnosis for 5–10 years regarded themselves as cancer patients compared to 40.2% of LTS who survived for more than ten years (*p* = 0.02). In LTS undergoing ongoing treatment, 80.3% regarded themselves as cancer patients.

### 3.2. Health Competence

Most LTS (80.8%) believed that physical exercise could positively influence the course of their cancer. However, 29.6% did not exercise regularly, while 22.9% did sports for 1–2 h per week, 19.6% for 2–4 h per week, and 16.7% for more than 4 h per week. There was no statistical difference in the physical activity levels between the LTS who had recurrent ovarian cancer compared to those without recurrent disease. Patients older than 60 years were less physically active: 34.5% >60 years compared to 22.2% ≤60 years did not exercise (*p* = 0.0001). Smoking was only reported by 10.1% of the participants. Nearly one third (32.2%) reported never drinking alcohol, while 6.0% drank more than four drinks per week. Almost two thirds of LTS (63.7%) were interested in studies concerning cancer survivorship. The online version of the questionnaire was more popular among the younger patients (*p* = 0.003). However, only 15 patients filled in the online questionnaire.

### 3.3. Follow-Up Care

Most LTS (94.2%) received regular follow-up care consisting of CA125 testing in 77.0%, clinical examination in 54.3%, transvaginal ultrasound in 55.1%, abdominal ultrasound in 43.9%, mammogram in 50.5%, and further radiological examinations such as CT or MRI scans in 53.4%. LTS without recurrences did not receive follow-up as frequently (7.7% without follow-up care vs. 2.9%, *p* = 0.0009). CA125 was tested more frequently in LTS with recurrences compared to LTS without recurrences (87.7% vs. 69.7%, *p* < 0.0001). Mammograms were more often undertaken in LTS without recurrence (53.6% vs. 46.8%, *p* = 0.03). Radiological examinations such as CT scans were performed in 70.7% of LTS with recurrences compared to 40.6% in those without (*p* < 0.0001).

## 4. Discussion

In this international survey ‘Expression VI—Carolin meets HANNA—Holistic Analysis of loNg-term survivors with ovariaN cAncer’, 1044 long-term survivors with ovarian cancer were recruited. Despite a very good or good health status in more than half of the patients (52.0%) and a very high frequency of regular oncological follow-up visits, long-term side effects like fatigue, lymphedema, and neurological symptoms were widespread.

The patients’ characteristics show that only 58.6% were diagnosed with advanced ovarian cancer (FIGO III/IV) compared to 41.4% with early-stage ovarian cancer. Nearly every survey participant received primary cytoreductive surgery, and platinum sensitivity was very high at 89.8%. Only 43.4% developed recurrent disease. This is very typical for long-term survivors with ovarian cancer and reflects the typical prognostic factors for ovarian cancer [6,7,8,18].

There was no difference between the lymphadenectomy rates between early- and advanced stage ovarian cancer patients. This could be due to the fact that all patients in this study received their initial diagnosis before 2017, which was before the LION study found that lymphadenectomy in patients with advanced-stage ovarian cancer with a complete macroscopic resection rate and normal lymph nodes is not necessary [19]. Our results indicating that general health status and distress are not associated with a history of lymphadenectomy and that lymphadenectomy is associated with symptoms such as polyneuropathy are in line with the LION study. Interestingly, a very high number of long-term survivors had surgery at recurrence (81.9%). This finding is in accordance with the DESKTOP III study, which showed the positive influence of recurrent ovarian cancer surgery on survival [20].

Follow-up care is usually carried out within the first five years after initial diagnosis. Patients are then often transferred to general physicians and gynecologists. Interestingly, in our cohort, most long-term survivors with a median survival time of eleven years after initial diagnosis still received regular follow-up care. Forty-six percent still experienced long-term side effects. These ranged from gastrointestinal problems to fatigue, lymphedema, and neurological long-term side effects. One of the reasons may be that long-term side effects are not routinely assessed within follow-up care or are no longer associated with cancer and cancer treatments. An improvement in side effects was associated with time of survival, underlining the importance of systematic assessment and treatment. However, such systematic assessment is time-consuming and expensive. Furthermore, long-term survivors frequently have long and complicated courses of disease with multiple surgical procedures and many lines of chemotherapy, so the side effects may become chronic. A survey of 129 ovarian cancer survivors showed that polyneuropathy remained high in a fifth of patients, even after twelve years [21]. The rates of lymphedema increased within the first years and reached a plateau of 20–25% of ovarian cancer survivors five to ten years after lymphadenectomy [22].

Patients regularly have more than one side effect at once such as LTS with fatigue, for example, who also suffer more frequently from neurological side effects and depression next to their fatigue. Therefore, it is difficult to treat long-term survivors within a routine ambulatory or clinical setting [23]. Special survivorship clinics beyond the typical five years involving an interdisciplinary team that is trained for the special needs of long-term survivors may be the key for improving long-term side effects and quality of life [24,25,26,27].

Both long-term side effects and quality of life are associated with survival, which is, of course, not only important for cancer survivors, but for all cancer patients [28,29,30,31]. The knowledge of long-term side effects can also help us to tailor treatment decisions in cancer patients and improve our consultations before, during, and after treatment. Our results demonstrate the high interest in studies and programs on cancer survivorship. This survey was one of the fundaments of the first survivorship clinic for long-term survivors with gynecological cancer in Germany (www.survivorship-clinic.de).

Within the follow-up care of long-term survivors, lifestyle factors should also be addressed [32,33,34]. Less than one third of the participants did not exercise regularly. It is known that regular exercise can not only improve long-term side effects such as fatigue and depression, but is also important with regard to the prevention of cardiovascular disease, which is very common in cancer patients and increases with survival years [35,36]. Arora et al. showed that death due to ovarian cancer is the main course of death within the first 15 years after initial diagnosis [37]. Other causes such as cardiovascular disease, falls, and secondary cancer become increasingly important causes of death within the years after diagnosis [37]. The median time from initial ovarian cancer diagnosis to secondary cancer was 6.5 years, proving again the importance of survivorship care longer than the typical first five years [38]. These findings emphasize the importance of implementing lifestyle factors and prevention in the long-term follow-up care of cancer patients [39,40].

Long-term survivors believe that physical exercise improves their course of the disease. More than half of the LTSs in this study changed their eating habits, demonstrating the interest and motivation of this cohort to improve their lifestyle factors. Interestingly, less than 20% of ovarian cancer survivors practiced high levels of physical activity in the Vivrovaire study compared to 70.4% of our cohort taking part in regular physical activity [41]. One explanation might be the long timeframe of eleven years after diagnosis in our cohort compared to a mean time of six years after the end of chemotherapy in the Vivrovaire study. We showed that health status improved with survival time, which might result in higher physical activity levels. Smoking and alcohol consumption did not play a significant role in this patient group compared to patients with other cancer entities.

Interim analyses of the Expression VI survey have influenced the GCIG consensus guideline on long-term survival with gynecological cancer [5]. This present analysis of 1044 long-term survivors with ovarian cancer has now confirmed the symptom checklist and clinical relevance of the consensus paper.

### Strengths and Limitations

This is the largest multicenter and international cohort of long-term survivors with ovarian cancer describing holistic patients’ characteristics, lifestyle issues, and medical symptoms. One limitation is that the data were derived from a prospective survey as opposed to a large, randomized clinical trial enrolling patients starting at initial cancer diagnosis. Long-term survival with gynecological cancer is defined as survival of at least five years after initial diagnosis. Unfortunately, only 30–40% of ovarian cancer patients become long-term survivors. A prospective multicenter trial for this patient cohort would be of great interest, but it would be very costly and would take immense effort to follow up the participants for more than 5 years. The median survival time of our 1044 recruited patients from 14 countries is 11 years, ranging from 5 to 46 years. A prospective trial starting at initial diagnosis is hardly possible for this cohort, but would be very valuable.

## 5. Conclusions

This present analysis confirms the high frequency of long-term side effects as well as constant levels of distress despite the high frequency of regular follow-up care. Our findings underline the clinical relevance of the symptom checklist within the GCIG consensus guideline on long-term survival with gynecological cancer. Specialized survivorship care with a focus on long-term side effects, lifestyle, and prevention should be offered beyond the typical five years of follow-up care.

## Figures and Tables

**Table 1:** Patients’ characteristics.

Age at initial diagnosis (years, *n* = 1041)	18–40	169 (16.2%)
	41–50	296 (28.4%)
	51–60	328 (31.5%)
	61–70	201 (19.3%)
	≥71	44 (4.2%)
FIGO stage (*n* = 500)	I	124 (24.8%)
	II	83 (16.6%)
	III	249 (49.8%)
	IV	44 (8.8%)
Primary surgery (*n* = 1038)	1029 (98.6%)	
Adjuvant chemotherapy (*n* = 1038)	955 (92.0%)	
Platinum sensitive (at 1st relapse) (*n* = 412)	370 (89.8%)	
Currently being treated (*n* = 930)	119 (12.7%)	
**Situation at recurrence**		
Recurrent disease (*n* = 1031)	447 (43.4%)	
Surgery at recurrence (*n* = 437)	358 (81.9%)	
Chemotherapy at recurrence (*n* = 426)	389 (91.3%)	

## Data Availability

The data are not yet publicly available due to the fact that the study is still recruiting.

## References

[1] DiSilvestro P., Banerjee S., Colombo N., Scambia G., Kim B.G., Oaknin A., Friedlander M., Lisyanskaya A., Floquet A., Leary A. (2023). Overall Survival with Maintenance Olaparib at a 7-Year Follow-Up in Patients with Newly Diagnosed Advanced Ovarian Cancer and a BRCA Mutation: The SOLO1/GOG 3004 Trial. J. Clin. Oncol..

[2] DiSilvestro P., Colombo N., Harter P., González-Martín A., Ray-Coquard I., Coleman R.L. (2021). Maintenance Treatment of Newly Diagnosed Advanced Ovarian Cancer: Time for a Paradigm Shift?. Cancers.

[3] Banerjee S., Moore K.N., Colombo N., Scambia G., Kim B.-G., Oaknin A., Friedlander M., Lisyanskaya A., Floquet A., Leary A. (2020). 811MO Maintenance olaparib for patients (pts) with newly diagnosed, advanced ovarian cancer (OC) and a BRCA mutation (BRCAm): 5-year (y) follow-up (f/u) from SOLO1. Ann. Oncol..

[4] Moore K., Colombo N., Scambia G., Kim B.G., Oaknin A., Friedlander M., Lisyanskaya A., Floquet A., Leary A., Sonke G.S. (2018). Maintenance Olaparib in Patients with Newly Diagnosed Advanced Ovarian Cancer. N. Engl. J. Med..

[5] Woopen H., Sehouli J., Davis A., Lee Y.C., Cohen P.A., Ferrero A., Gleeson N., Jhingran A., Kajimoto Y., Mayadev J. (2022). GCIG-Consensus guideline for Long-term survivorship in gynecologic Cancer: A position paper from the gynecologic cancer Intergroup (GCIG) symptom benefit committee. Cancer Treat. Rev..

[6] Baum J., Braicu E.I., Hunsicker O., Vergote I., Concin N., Van Nieuwenhuysen E., Feldheiser A., Achimas-Cadariu P., Darb-Esfahani S., Berger A. (2021). Impact of clinical factors and surgical outcome on long-term survival in high-grade serous ovarian cancer: A multicenter analysis. Int. J. Gynecol. Cancer.

[7] Cress R.D., Chen Y.S., Morris C.R., Petersen M., Leiserowitz G.S. (2015). Characteristics of Long-Term Survivors of Epithelial Ovarian Cancer. Obstet. Gynecol..

[8] Hoppenot C., Eckert M.A., Tienda S.M., Lengyel E. (2018). Who are the long-term survivors of high grade serous ovarian cancer?. Gynecol. Oncol..

[9] Garsed D.W., Pandey A., Fereday S., Kennedy C.J., Takahashi K., Alsop K., Hamilton P.T., Hendley J., Chiew Y.-E., Traficante N. (2022). The genomic and immune landscape of long-term survivors of high-grade serous ovarian cancer. Nat. Genet..

[10] Javellana M., Hoppenot C., Lengyel E. (2019). The road to long-term survival: Surgical approach and longitudinal treatments of long-term survivors of advanced-stage serous ovarian cancer. Gynecol. Oncol..

[11] Winter W.E., Maxwell G.L., Tian C., Carlson J.W., Ozols R.F., Rose P.G., Markman M., Armstrong D.K., Muggia F., McGuire W.P. (2007). Gynecologic Oncology Group Study. Prognostic factors for stage III epithelial ovarian cancer: A Gynecologic Oncology Group Study. J. Clin. Oncol..

[12] Kajiyama H., Shibata K., Mizuno M., Umezu T., Suzuki S., Yamamoto E., Fujiwara S., Kawai M., Nagasaka T., Kikkawa F. (2012). Long-term clinical outcome of patients with recurrent epithelial ovarian carcinoma: Is it the same for each histological type?. Int. J. Gynecol. Cancer.

[13] Westin S.N., Sun C.C., Tung C.S., Lacour R.A., Meyer L.A., Urbauer D.L., Frumovitz M.M., Lu K.H., Bodurka D.C. (2016). Survivors of gynecologic malignancies: Impact of treatment on health and well-being. J. Cancer Surviv..

[14] Matsuoka H., Nakamura K., Matsubara Y., Ida N., Saijo M., Ogawa C., Masuyama H. (2018). The Influence of Chemotherapy-Induced Peripheral Neuropathy on Quality of Life of Gynecologic Cancer Survivors. Int. J. Gynecol. Cancer.

[15] Rietveld M.J.A., Husson O., Vos M.C.C., van de Poll-Franse L.V., Ottevanger P.B.N., Ezendam N.P.M. (2019). Presence of gastro-intestinal symptoms in ovarian cancer patients during survivorship: A cross-sectional study from the PROFILES registry. Support. Care Cancer.

[16] Lockwood-Rayermann S. (2006). Survivorship issues in ovarian cancer: A review. Oncol. Nurs. Forum.

[17] De Rosa N., Della Corte L., Giannattasio A., Giampaolino P., Di Carlo C., Bifulco G. (2021). Cancer-related cognitive impairment (CRCI), depression and quality of life in gynecological cancer patients: A prospective study. Arch. Gynecol. Obstet..

[18] Dao F., Schlappe B.A., Tseng J., Lester J., Nick A.M., Lutgendorf S.K., McMeekin S., Coleman R.L., Moore K.N., Karlan B.Y. (2016). Characteristics of 10-year survivors of high-grade serous ovarian carcinoma. Gynecol. Oncol..

[19] Harter P., Sehouli J., Lorusso D., Reuss A., Vergote I., Marth C., Kim J.-W., Raspagliesi F., Lampe B., Aletti G. (2019). A Randomized Trial of Lymphadenectomy in Patients with Advanced Ovarian Neoplasms. N. Engl. J. Med..

[20] Harter P., Sehouli J., Vergote I., Ferron G., Reuss A., Meier W., Greggi S., Mosgaard B.J., Selle F., Guyon F. (2021). Randomized Trial of Cytoreductive Surgery for Relapsed Ovarian Cancer. N. Engl. J. Med..

[21] Ezendam N.P.M., Pijlman B., Bhugwandass C., Pruijt J.F.M., Mols F., Vos M.C., Pijnenborg J.M., van de Poll-Franse L.V. (2014). Chemotherapy-induced peripheral neuropathy and its impact on health-related quality of life among ovarian cancer survivors: Results from the population-based PROFILES registry. Gynecol. Oncol..

[22] Hareyama H., Hada K., Goto K., Watanabe S., Hakoyama M., Oku K., Hayakashi Y., Hirayama E., Okuyama K. (2015). Prevalence, classification, and risk factors for postoperative lower extremity lymphedema in women with gynecologic malignancies: A retrospective study. Int. J. Gynecol. Cancer.

[23] Gallego A., Martínez B., Ghanem I., Cantero J.M., Espinosa E., Castelo B., Zamora P., Ruiz-Gimenez L., Redondo A., Feliu J. (2021). Cancer survivors referred to a long-term survivorship outpatient service within academic medical oncology: Descriptive study. J. Cancer Surviv..

[24] Lokich E. (2019). Gynecologic Cancer Survivorship. Obstet. Gynecol. Clin. N. Am..

[25] Hill R.E., Wakefield C.E., Cohn R.J., Fardell J.E., Brierley M.E., Kothe E., Jacobsen P.B., Hetherington K., Mercieca-Bebber R. (2020). Survivorship Care Plans in Cancer: A Meta-Analysis and Systematic Review of Care Plan Outcomes. Oncologist.

[26] De Rooij B.H., Thomas T.H., Post K.E., Flanagan J., Ezendam N.P.M., Peppercorn J., Dizon D.S. (2018). Survivorship care planning in gynecologic oncology-perspectives from patients, caregivers, and health care providers. J. Cancer Surviv..

[27] Oeffinger K.C., Argenbright K.E., Levitt G.A., McCabe M.S., Anderson P.R., Berry E., Maher J., Merrill J., Wollins D.S. (2014). Models of cancer survivorship health care: Moving forward. Am. Soc. Clin. Oncol. Educ. Book.

[28] Wenzel L., Osann K., McKinney C., Cella D., Fulci G., Scroggins M.J., Lankes H., Wang V., Nephew K.P., Maxwell G.L. (2021). Quality of Life and Adverse Events: Prognostic Relationships in Long-Term Ovarian Cancer Survival. J. Natl. Cancer Inst..

[29] Ashing-Giwa K.T., Lim J.W., Tang J. (2010). Surviving cervical cancer: Does health-related quality of life influence survival?. Gynecol. Oncol..

[30] Woopen H., Richter R., Inci G., Alavi S., Chekerov R., Sehouli J. (2020). The prognostic and predictive role of pain before systemic chemotherapy in recurrent ovarian cancer: An individual participant data meta-analysis of the North-Eastern German Society of Gynecological Oncology (NOGGO) of 1226 patients. Support. Care Cancer.

[31] Roncolato F.T., Gibbs E., Lee C.K., Asher R., Davies L.C., Gebski V.J., Friedlander M., Hilpert F., Wenzel L., Stockler M. (2017). Quality of life predicts overall survival in women with platinum-resistant ovarian cancer: An AURELIA substudy. Ann. Oncol..

[32] Denlinger C.S., Sanft T., Moslehi J.J., Overholser L., Armenian S., Baker K.S., Broderick G., Demark-Wahnefried W., Friedman D.L., Goldman M. (2020). NCCN Guidelines Insights: Survivorship, Version 2.2020. J. Natl. Compr. Canc Netw..

[33] Thomson C.A., Crane T.E., Miller A., Gold M.A., Powell M., Bixel K., Van Le L., DiSilvestro P., Ratner E., Lele S. (2023). Lifestyle intervention in ovarian cancer enhanced survival (LIVES) study (NRG/GOG0225): Recruitment, retention and baseline characteristics of a randomized trial of diet and physical activity in ovarian cancer survivors. Gynecol. Oncol..

[34] Smits A., Lopes A., Das N., Bekkers R., Massuger L., Galaal K. (2015). The effect of lifestyle interventions on the quality of life of gynaecological cancer survivors: A systematic review and meta-analysis. Gynecol. Oncol..

[35] Sturgeon K.M., Deng L., Bluethmann S.M., Zhou S., Trifiletti D.M., Jiang C., Kelly S.P., Zaorsky N.G. (2019). A population-based study of cardiovascular disease mortality risk in US cancer patients. Eur. Heart J..

[36] Campbell K.L., Winters-Stone K.M., Wiskemann J., May A.M., Schwartz A.L., Courneya K.S., Zucker D.S., Matthews C.E., Ligibel J.A., Gerber L.H. (2019). Exercise Guidelines for Cancer Survivors: Consensus Statement from International Multidisciplinary Roundtable. Med. Sci. Sports Exerc..

[37] Arora N., Talhouk A., McAlpine J.N., Law M.R., Hanley G.E. (2019). Causes of death among women with epithelial ovarian cancer by length of survival post-diagnosis: A population-based study in British Columbia, Canada. Int. J. Gynecol. Cancer.

[38] Woopen H., Rolf C., Braicu E.I., Buttmann-Schweiger N., Barnes B., Baum J., Pietzner K., Kraywinkel K., Sehouli J. (2021). Secondary malignancies in long-term ovarian cancer survivors: Results of the ‘Carolin meets HANNA’ study. Int. J. Gynecol. Cancer.

[39] Monllor-Tormos A., García-Vigara A., Morgan O., García-Pérez M.Á., Mendoza N., Tarín J.J., Cano A. (2023). Mediterranean diet for cancer prevention and survivorship. Maturitas.

[40] Vaz-Luis I., Masiero M., Cavaletti G., Cervantes A., Chlebowski R.T., Curigliano G., Felip E., Ferreira A.R., Ganz P.A., Hegarty J. (2022). ESMO Expert Consensus Statements on Cancer Survivorship: Promoting high-quality survivorship care and research in Europe. Ann. Oncol..

[41] Joly F., Ahmed-Lecheheb D., Kalbacher E., Heutte N., Clarisse B., Grellard J.M., Gernier F., Berton-Rigaud D., Tredan O., Fabbro M. (2019). Long-term fatigue and quality of life among epithelial ovarian cancer survivors: A GINECO case/control VIVROVAIRE I study. Ann. Oncol..

